# The effects of flow settings during high-flow nasal cannula support for adult subjects: a systematic review

**DOI:** 10.1186/s13054-023-04361-5

**Published:** 2023-02-28

**Authors:** Jie Li, Fai A. Albuainain, Wei Tan, J. Brady Scott, Oriol Roca, Tommaso Mauri

**Affiliations:** 1 Division of Respiratory Care, Department of Cardiopulmonary Sciences, Rush University, 600 S Paulina St, Suite 765, Chicago, IL 60612 USA; 2 Department of Respiratory Care, Imam Abdulrahman Bin Faisal University, Dammam, Saudi Arabia; 3 Department of Respiratory and Critical Care Medicine, The First Affiliated Hospital of China Medical University, Shenyang, China; 4 Servei de Medicina Intensiva, Parc Taulí Hospital Universitari, Sabadell, Spain; 5 Centro de Investigacion Biomedica en Red de Enfermedades Respiratorias (CIBERES), Instituto de Salud Carlos III, Madrid, Spain; 6 Departament de Medicina, Universitat Autònoma de Barcelona, Bellaterra, Spain; 7 Department of Anesthesia, Critical Care and Emergency, Fondazione IRCCS Ca’ Granda Ospedale Maggiore Policlinico, Milan, Italy; 8 Department of Pathophysiology and Transplantation, University of Milan, Milan, Italy

**Keywords:** High-flow nasal cannula, Oxygen therapy, Flow settings, Peak inspiratory flow, Oxygenation, Ventilation distribution, Patient self-inflicted lung injury

## Abstract

**Background:**

During high-flow nasal cannula (HFNC) therapy, flow plays a crucial role in the physiological effects. However, there is no consensus on the initial flow settings and subsequent titration. Thus, we aimed to systematically synthesize the effects of flows during HFNC treatment.

**Methods:**

In this systematic review, two investigators independently searched PubMed, Embase, Web of Science, Scopus, and Cochrane for in vitro and in vivo studies investigating the effects of flows in HFNC treatment published in English before July 10, 2022. We excluded studies that investigated the pediatric population (< 18 years) or used only one flow. Two investigators independently extracted the data and assessed the risk of bias. The study protocol was prospectively registered with PROSPERO, CRD42022345419.

**Results:**

In total, 32,543 studies were identified, and 44 were included. In vitro studies evaluated the effects of flow settings on the fraction of inspired oxygen (F_I_O_2_), positive end-expiratory pressure, and carbon dioxide (CO_2_) washout. These effects are flow-dependent and are maximized when the flow exceeds the patient peak inspiratory flow, which varies between patients and disease conditions. In vivo studies report that higher flows result in improved oxygenation and dead space washout and can reduce work of breathing. Higher flows also lead to alveolar overdistention in non-dependent lung regions and patient discomfort. The impact of flows on different patients is largely heterogeneous.

**Interpretation:**

Individualizing flow settings during HFNC treatment is necessary, and titrating flow based on clinical findings like oxygenation, respiratory rates, ROX index, and patient comfort is a pragmatic way forward.

**Supplementary Information:**

The online version contains supplementary material available at 10.1186/s13054-023-04361-5.

## Introduction

The use of high-flow nasal cannula (HFNC) in critical care areas has increased over the past few years, particularly during the COVID-19 pandemic [1]. HFNC has been shown to effectively reduce intubation rates for patients with acute hypoxemic respiratory failure (AHRF) [2] and prevent post-extubation respiratory failure [3]. It may also be non-inferior to noninvasive ventilation to prevent reintubation for patients with chronic obstructive pulmonary disease (COPD) [4, 5]. Improved patient outcomes associated with HFNC are due to its physiological effects, such as improvement in oxygenation [6–11], efficiency of ventilation [6–8, 11–24], reduction of work of breathing (WOB) [7, 11, 12, 24], avoidance of patient self-inflicted lung injury, and improvement in patient comfort and tolerance [25]. HFNC washes out upper airway dead space, and its effects are maximized when the delivered gas flow meets or exceeds the patient peak inspiratory flow, resulting in a stable fraction of inspired oxygen (F_I_O_2_) and a level of positive end-expiratory pressure (PEEP) [6, 26]. Thus, flow settings play a vital role during HFNC oxygen therapy [27].

In recent years, significant efforts have been made to investigate the effects of flow settings during HFNC therapy for various patient populations. However, no consensus has been reached on the most effective initial flow setting and its subsequent titration. Therefore, we systematically reviewed the available evidence regarding the physiological and clinical effects of different flow settings during HFNC therapy for adult subjects, aiming to provide evidence-based guidance on optimal HFNC flow settings for various clinical conditions.

## Literature search strategy and results

A literature search was conducted independently by two investigators in PubMed, Embase, Web of Science, Scopus, and Cochrane for articles published before July 10, 2022, using the following keywords: (“high-flow nasal cannul*” OR “high flow cannul*” OR “high flow oxygen therapy” OR “high flow oxygen” OR “high flow therapy” OR “HFNC” OR “nasal high flow” OR “NHF”) AND (“flow”) AND (“adult”). The search was limited to papers published in English. Original studies investigating more than one HFNC flow setting were included. Studies that only included pediatric populations, used only one flow during HFNC treatment, review articles, letters, abstracts, and editorials were excluded. Study titles and abstracts were initially screened, and full texts were subsequently reviewed to select studies included in this review. The review protocol was prospectively registered with PROSPERO, CRD42022345419. Two investigators independently extracted the data and assessed the risk of bias using the Cochrane collaboration risk of bias tool for RCTs. The Newcastle–Ottawa Scale was used to assess non-randomized trials. Any disagreement regarding study selection, data extraction, or quality assessments was resolved by a consensus discussion with the third investigator.

**Fig. 1:**
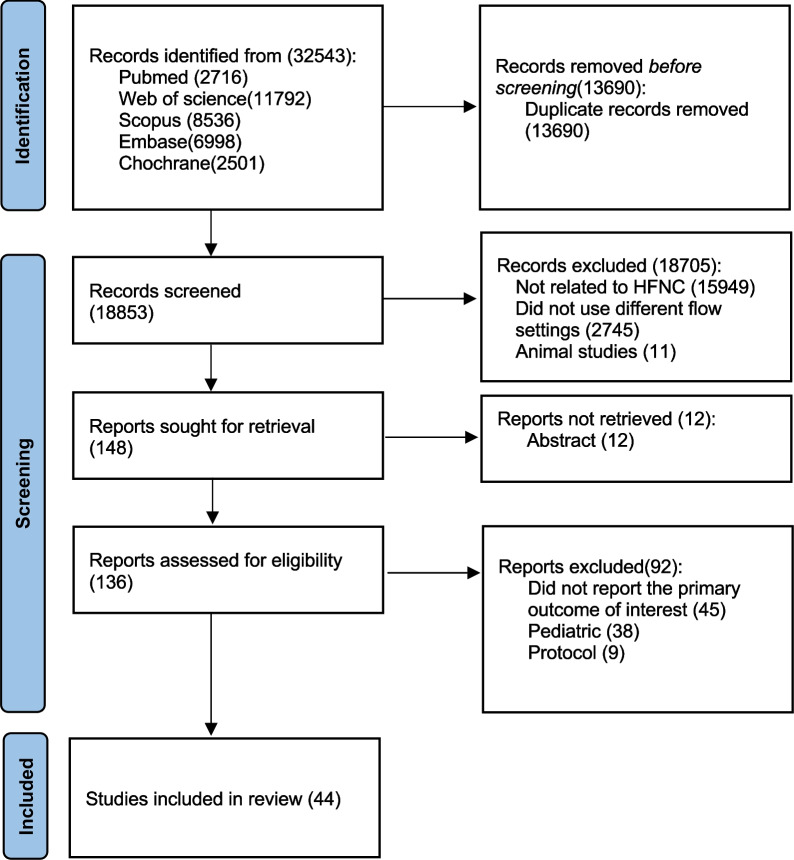
Study flow diagram. HFNC, high-flow nasal cannula

A total of 32,543 studies were identified, and 32,395 studies were excluded for reasons displayed in Fig. 1. One hundred and forty-eight full-text articles were assessed for eligibility, and 44 studies were finally included, of which 11 were in vitro studies [26, 28–37], 2 combined in vitro and in vivo studies to investigate patients with AHRF [6, 14], 13 studies investigated healthy individuals [13, 15–20, 38–43], 9 investigated patients with AHRF [7–10, 27, 44–46, 50], 5 studies examined patients with COPD [11, 12, 21–23], 1 study investigated both AHRF and COPD patients [24], and 3 investigated patients during procedural sedation [47–49]. Among the in vivo studies, only four were randomized controlled trials [27, 47–49], while 18 were randomized crossover studies [7, 8, 10, 12, 13, 16, 17, 22–24, 38, 40–43, 45, 46, 50]. None of the included randomized trials had incomplete outcome data reporting but 10 did not have registration [7, 8, 10, 13, 41, 43, 46, 48–50]. All of them had a clear description of random sequence generation, but only five explained the study allocation concealment [13, 16, 22, 27, 48]. Due to the nature of HFNC flows in conscious patients, blinding participants and/or the treating clinicians was not possible. No obvious publication bias was observed among the randomized controlled or crossover trials (Additional file 1: Figs. S1, appendix p2) and non-randomized trials (Additional file 1: Table S1, appendix p3).

## Subject peak inspiratory flow during tidal breathing

HFNC aims to provide a flow that meets or exceeds the patient peak tidal inspiratory flow (PTIF) [51]. Several studies have reported PTIF in different populations (Additional file 1: Table S2, appendix p4) [6, 13, 44, 52–54].

### Healthy individuals

Healthy adult volunteer PTIF has been reported in two studies [13, 52]. Ritchie et al. [13] reported PTIFs while study participants were at rest and during exercise. PTIF during exercise was higher (119.9 ± 20.0 vs 27.9 ± 9.2 L/min). Moreover, PTIF increased as exercise intensity increased [52].

### Patients with pulmonary disease

The PTIF in adult patients with pulmonary disease, especially for those with respiratory failure, is slightly higher than PTIF in healthy individuals. Small variations in PTIF have been noted between different diseases (Additional file 1: Table S2, appendix p4). The mean PTIF in patients with AHRF was reported to be 34 ± 9 L/min [6]. Similar median PTIF values were observed in patients with stable asthma and COPD [53]. In stable tracheostomized patients, the mean PTIF was 30 (27, 32) L/min when measured inside the trachea [54]. Interestingly, Butt et al. [44] measured PTIF in intubated patients on a mechanical ventilator with pressure support of zero and zero-PEEP. Their measured PTIF was 60 (40, 80) L/min, significantly higher than the PTIF found in non-intubated spontaneously breathing patients. This difference might be caused by the need to overcome the resistance of the endotracheal tube or by a more severe respiratory condition.

## In vitro evidence of flow settings during HFNC treatment

**Fig. 2:**
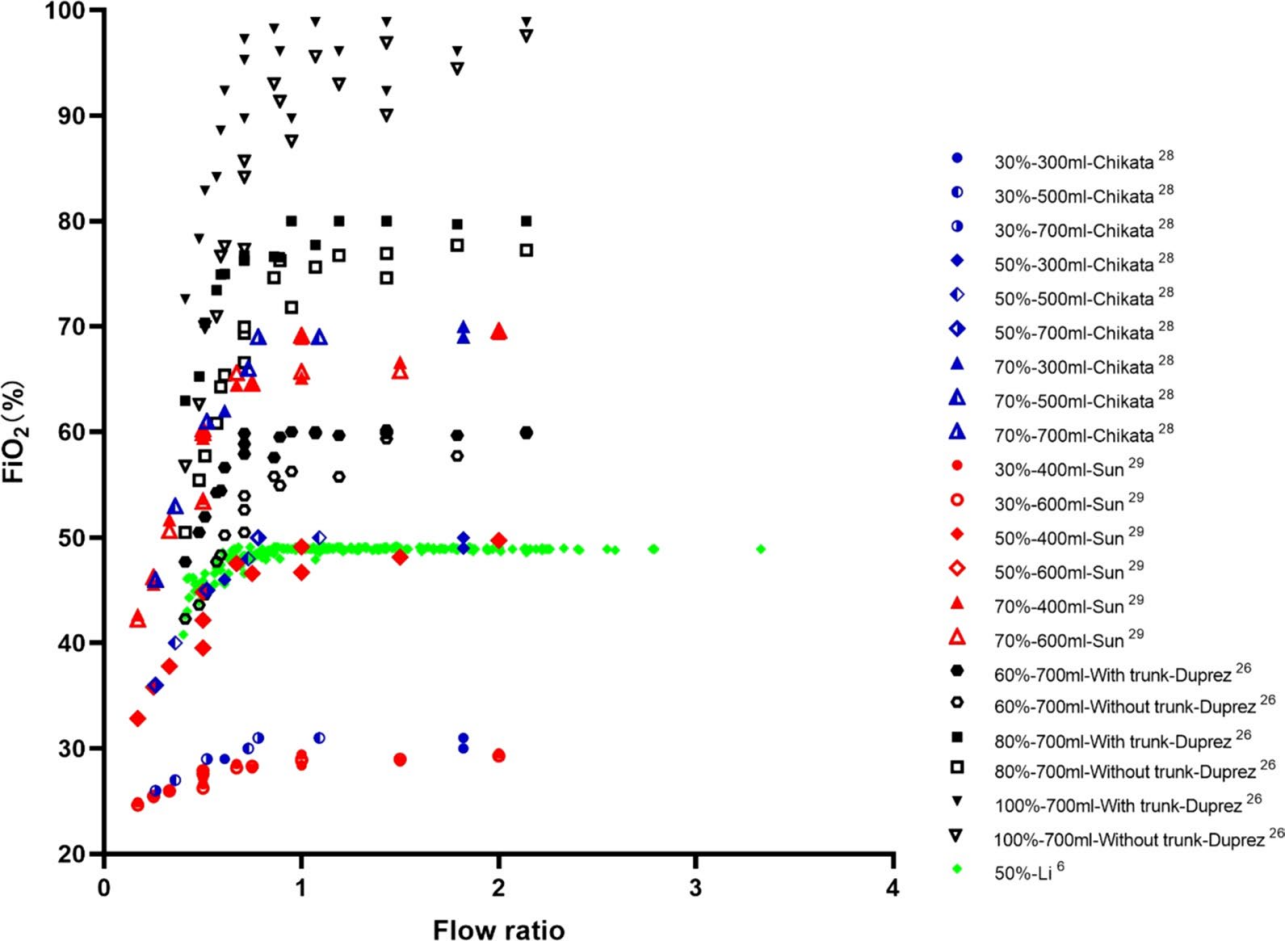
Relationship between F_I_O_2_ and flow ratio of HFNC flow to peak inspiratory flow during tidal breathing. F_I_O_2_, fraction of inspired oxygen; HFNC, high-flow nasal cannula

**Fig. 3:**
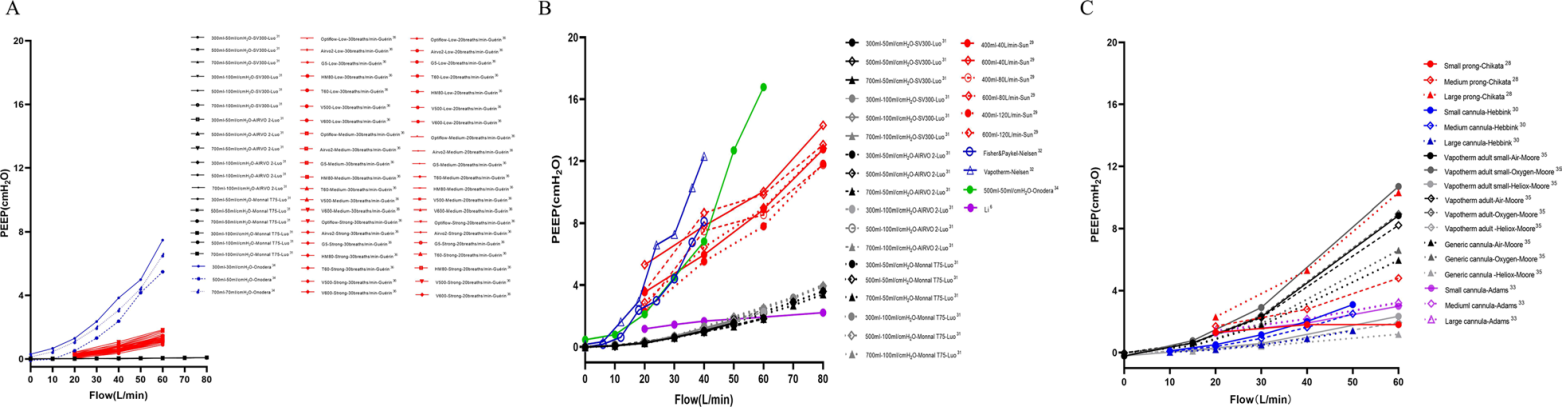
Effects of mouth status (open- vs closed-mouth breathing) (**A**, **B**), lung compliance (**B**), gas type (**C**), and nasal prong size (**C**) on PEEP levels. PEEP, positive end-expiratory pressure; HFNC, high-flow nasal cannula

**Fig. 4:**
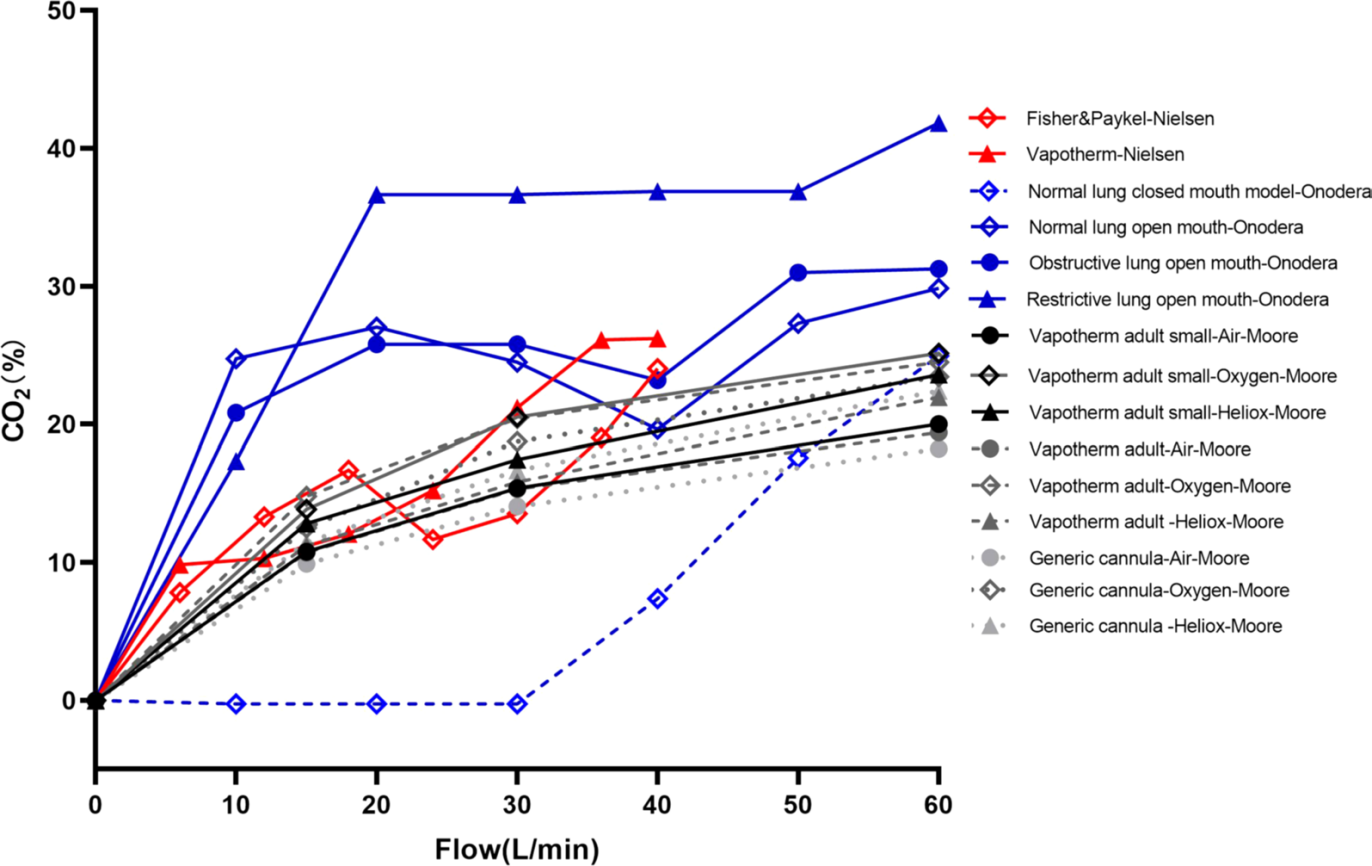
Relationship between CO_2_ clearance and HFNC flow settings. CO_2_, carbon dioxide; HFNC, high-flow nasal cannula

Multiple bench studies have been conducted to evaluate the effects of HFNC flows on tracheal F_I_O_2_, [6, 26, 28, 29] PEEP [6, 28–36], and dead space clearance [14, 32, 34, 35, 37]. When HFNC flow is lower than PTIF, the F_I_O_2_ in the trachea is lower than the set F_I_O_2_ (Fig. 2) [6, 26, 28, 29]. This can be explained by air entrainment occurring in the upper airway, which dilutes the concentration of delivered oxygen [26]. When HFNC flow exceeds PTIF, studies have shown that a certain level of PEEP is indeed generated [6], with a quadratic correlation between HFNC flows and PEEP levels [33, 35]. That said, multiple factors affect PEEP level besides flow settings and include mouth status (open- vs closed-mouth breathing) (Fig. 3A, B) [31, 34], lung compliance (Fig. 3B) [31], gas type (Fig. 3C) [35], and nasal prong size (Figs. 3C) [28, 30, 33, 35]. Of these, it appears that mouth status is the most impactful variable on PEEP generation [31, 32]. During closed-mouth breathing, PEEP can be as high as 14.31 ± 1.33 cmH_2_O when flows of 80 L/min are set in adults [29]. When the mouth is open, however, the PEEP level drops to almost zero [31]. Additionally, PEEP levels are lower in stiffer lungs, where PEEP is needed most [31]. Lighter gases, such as heliox [35], and smaller nasal prongs have also been reported to generate lower PEEP [14, 35]. Finally, high HFNC flows appear to clear CO_2_ in less time, even when respiratory rates remain constant (Fig. 4) [32, 34, 35]. Overall, when flows are set to exceed PTIF, HFNC can produce a certain amount of PEEP, stabilize F_I_O_2_ delivery, and wash out anatomic dead space. However, mouth open/close status, lung compliance, gas type, and prong size also impact HFNC effects.

## Different flow settings in adult subjects

During HFNC oxygen therapy, flow settings have been shown to have a significant impact on short-term clinical outcomes in 32 studies [6–24, 38–50] and long-term outcomes in one study [27].

### Healthy individuals

#### Ventilation

Similar to in vitro studies, the physiological effects of HFNC are found to be flow-dependent (Table 1) [13, 15–20, 38–40]. As HFNC flows increase from 0 to 40 L/min, tidal volume (Vt) increases [15–17, 38] and respiratory rate (RR) decreases [13, 15–20]. However, Okuda et al. reported no change in minute ventilation between HFNC flows of 0 and 50 L/min [15], and Parke et al. [18] reported that RR plateaued with HFNC flows > 40 L/min.

**Table 1:** Physiological effects of flow settings during HFNC treatment for healthy volunteers

Author/year	Study design	*N*	Position	Breathing pattern	Flow (L/min)	RR (breaths/min)	$\Delta$EELI_glob_ (units)	VT (ml)	PEEP (cmH_2_O)	PTP (cmH_2_O s/min)	Others
Okuda et al., 2017 [15]	Randomized crossover	10	Supine 30°	Not specified	0	15.1 ± 2.1	NA	685.6 ± 236.6	NA	NA	MV: 10.1 ± 3.0 $\Delta$ Pes:4.2 ± 1.9
					30	11.6 ± 3.1	NA	929.8 ± 434.7	NA	NA	MV: 10.1 ± 3.9 $\Delta$ Pes: 6.9 ± 2.9
					50	11.1 ± 2.3	NA	968.8 ± 451.1	NA	NA	MV: 10.1 ± 4.0 $\Delta$ Pes: 7.48 ± 3.1
Moigne et al. 2021 [38]	Prospective	10	Supine 30°	RMO	0	NA	NA	526 (503, 604)	NA	NA	NA
				RMC		NA	NA	529 (480,726)	NA	NA	NA
				HMO		NA	NA	540 (356, 663)	NA	NA	NA
				HMC		NA	NA	417 (331, 553)	NA	NA	NA
				RMO	30	NA	NA	688 (629, 844)	NA	NA	NA
				RMC		NA	NA	815 (511, 959)	NA	NA	NA
				HMO		NA	NA	633 (538, 678)	NA	NA	NA
				HMC		NA	NA	516 (405, 698)	NA	NA	NA
				RMO	60	NA	NA	753 (650, 1208)	NA	NA	NA
				RMC		NA	NA	913 (680, 1166)	NA	NA	NA
				HMO		NA	NA	664 (634, 1046)	NA	NA	NA
				HMC		NA	NA	696 (351, 896)	NA	NA	NA
Plotnikow et al. 2018 [19]	Prospective	16	Supine	RMC	0	16 ± 2.6	Baseline	NA	NA	NA	NA
			Setting 45°			15 ± 3.6	1.05 (0.72, 1.34)	NA	NA	NA	NA
					30	11 ± 3.6	1.12 (0.8, 2.01)	NA	NA	NA	NA
					50	9 ± 2.8	1.44 (1.05, 2.16)	NA	NA	NA	NA
Ritchie et al. 2011 [13]	Prospective	10	Not specified	RMC	30	8.4 (4,15)^b^	NA	NA	2.5 (0.8, 4.7)^b^	NA	NA
					40	6.8 (4,7)^b^	NA	NA	4.0 (2.4, 7.8)^b^	NA	NA
					50	7.6 (4.9, 8.0)^b^	NA	NA	5.2 (3.5, 7.0)^b^	NA	NA
				RMO	30	10 (6.9, 19)^b^	NA	NA	0.1 (− 0.2, 1)^b^	NA	NA
					40	9.9 (5.5, 19)^b^	NA	NA	0.8 (− 0.8, 1.2)^b^	NA	NA
					50	11.3 (7, 21.5)^b^	NA	NA	1.1 (− 0.9, 2.4)^b^	NA	NA
Parke et al. 2015 [18]	Prospective	15	Not specified	RMC	0	13.8 (9.8, 17.9)^b^	Baseline	NA	0.5 ± 0.3^a^	NA	NA
					30	8.9 (5.8,11.9)^b^	0.5 ± 0.3^a,c^	NA	2.7 ± 0.7^a^	NA	NA
					40	7.7 (4.5, 11.1)^b^	0.5 ± 0.4^a,c^	NA	3.8 ± 0.8^a^	NA	NA
					50	7.3 (4.4, 10.3)^b^	0.7 ± 0.5^a,c^	NA	4.9 ± 1.1^a^	NA	NA
					60	7.4 (3.8, 11.1)^b^	0.9 ± 0.6^a,c^	NA	6.1 ± 1.4^a^	NA	NA
					70	6.9 (3.2,10.7)^b^	1.0 ± 0.8^a,c^	NA	7.6 ± 1.5^a^	NA	NA
					80	7.7 (3.6, 11.8)^b^	1.2 ± 0.8^a,c^	NA	9 ± 1.9^a^	NA	NA
					90	6.9 (3.3, 10.4)^b^	1.4 ± 0.8^a,c^	NA	10.1 ± 2.1^a^	NA	NA
					100	7.5 (2.6,12.5)^b^	1.6 ± 0.8^a,c^	NA	11.9 ± 2.7^a^	NA	NA
Delorme et al. 2020 [16]	Randomized crossover	10	Semi-recumbent position	RMC	0	16 (15, 18)	NA	337 (272, 443)	NA	NA	Comfort: 10 (10, 10) V_A_: 3,343; VD/VT: 45 Dead space ventilation: 2431 Dead space washout: 0
					5	13 (12, 15)	NA	358 (287, 458)	NA	NA	Comfort: 9 (6, 10) V_A_: 3,343; VD/VT: 39 Dead space ventilation: 1823 Dead space washout: 863
					20	10 (10, 13)	NA	448 (345, 580)	NA	NA	Comfort: 10 (9, 10) V_A_: 3,343; VD/VT: 38 Dead space ventilation: 1546 Dead space washout: 1140
					40	9 (8, 12)	NA	450 (307, 480)	NA	NA	Comfort: 9 (7, 10) V_A_: 3,343; VD/VT: 34 Dead space ventilation: 1276 Dead space washout: 1191
					60	8 (7, 10)	NA	520 (470, 626)	NA	NA	Comfort: 8 (8, 9) V_A_: 3,343; VD/VT: 31 Dead space ventilation: 1276 Dead space washout: 1160
Garofalo et al. 2019 [20]	Prospective	14	Semi-recumbent position	RMC	0	15 (14,16)	NA	NA	0^b^	NA	Comfort: 10 (10,10 PEIP: 0^b^
					30	15 (14,16)	NA	NA	2.8 (0.8, 7.8)^b^	NA	Comfort: 10 (10,10) PEIP: 0.7 (0.3, 0.7)^b^
					40	14 (12,14)	NA	NA	6 (3.2, 11)^b^	NA	Comfort: 10 (10,10) PEIP: 0.8 (0.6, 1.3)^b^
					50	12 (12, 13)	NA	NA	7.6 (4.1, 12)^b^	NA	Comfort: 9 (9,10) PEIP: 1.1 (0.7, 1.5)^b^
				RMO	0	NA	NA	NA	0^b^	NA	PEIP: 0^b^
					30	NA	NA	NA	0.7 (0.5, 2.5)^b^	NA	PEIP: 0.1 (0, 0.4)^b^
					40	NA	NA	NA	1.7 (0.3, 5)^b^	NA	PEIP: 0.3 (0, 0.3)^b^
					50	NA	NA	NA	2.3 (0.7, 3.9)^b^	NA	PEIP: 0.3 (0, 0.6)^b^
Vieira et al. 2022 [17]	Randomized crossover	10	Not specified	RMC	20	9.0 (7.3, 11.7)	90 (43, 160)	458 (364, 557)	1.2 (0.8, 1.3)	94.1 (79.3, 114.5)	MV: 4.4 (3.2, 4.9 T_i_:T_tot_: 0.31 ± 0.05
					40	7.2 (5.9, 9.9)	160 (96, 178)	447 (368, 787)	3.6 (2.8, 4.6)	112.5 (86, 127)	MV: 3.9 (2.7, 4.2) T_i_:T_tot_: 0.26 ± 0.06
					60	8.5 (5.0, 12.4)	230 (165, 344)	431 (229, 760)	6.78 (5.0, 7.9)	124.8 (72, 138)	MV: 3.2 (2. 7,4.6) T_i_:T_tot_: 0.23 ± 0.07
				RMO	20	11.6 (9.4, 14.2)	70 (15, 138)	421 (341, 517)	0.13 (0.06, 0.2)	70.0 (50.6, 103)	MV: 5.2 (4.3, 6.1) T_i_:T_tot_: 0.33 ± 0.09
					40	11.6 (8.9, 12.6)	92 (36, 151)	459 (388, 502)	0.55 (0.3, 0.7)	78.5 (66, 102)	MV:5.2 (4.1–6.7) T_i_:T_tot_: 0.3 ± 0.07
					60	12.7 (10.4,15.8)	202 (115, 233)	504 (385, 657)	0.83 (0.6, 1.2)	90.4 (70, 111)	MV: 6.0 (4.8, 9.1) T_i_:T_tot_: 0.3 ± 0.07
Groves et al. 2007 [39]	Prospective	10	Setting position	RMC	0	NA	NA	NA	0.8 (0.5, 1.3)	NA	MIP: − 1.1 (− 2.0, − 0.6)
					10	NA	NA	NA	1.7 (1.2, 2.3)	NA	MIP: − 0.8 (− 1.1, − 0.3)
					20	NA	NA	NA	2.9 (2.2, 2.7)	NA	MIP: − 0.2 (− 0.9, 0.2)
					40	NA	NA	NA	5.5 (4.1, 7.2)	NA	MIP:1.1 (− 0.1, 1.9)
					60	NA	NA	NA	7.4 (5.4, 8.8)	NA	MIP: 1.6 (0.8, 2.7)
				RMO	0	NA	NA	NA	0.3 (0.3, 0.5)	NA	MIP: − 0.6 (− 1.1, − 0.4)
					10	NA	NA	NA	0.7 (0.6, 0.9)	NA	MIP: − 0.2 (− 0.8, 0.1)
					20	NA	NA	NA	1.4 (1.3, 1.8)	NA	MIP: − 0.2 (− 0.9, 0.1)
					40	NA	NA	NA	2.2 (2.0, 2.5)	NA	MIP:0.1 (− 0.2, 0.4)
					60	NA	NA	NA	2.7 (2.4, 3.1)	NA	MIP:0.5 (0.2, 0.7)
Möller et al. 2017 [40]	Randomized crossover	10	Not specified	RMC	15	NA	NA	NA	NA	NA	Clearance half-time in trachea: 23.73 ± 6.63
					30	NA	NA	NA	NA	NA	Clearance half-time in trachea:14.3 ± 13.43
					45	NA	NA	NA	NA	NA	Clearance half-time in trachea:10.53 ± 9.85

*RR* respiratory rate*,* $\Delta$ *EELI*_*glob*_ global change in end-expiratory lung impedance measured by electrical impedance tomography, *VT* tidal volume, *PEEP* positive end-expiratory pressure measured by pharyngeal or tracheal catheter, *MV* minute ventilation (L/min), *PTP* esophageal pressure-time product per minute (cmH_2_O·s/min), $\Delta$ *Pes* esophageal pressure swings (cmH_2_O), *RMO* regular respiratory rate breathing with mouth opened, *RMC* regular respiratory rate breathing with mouth closed, *HMO* high respiratory rate breathing with mouth opened, *HMC* high respiratory rate breathing with mouth closed*. Ti:T*_*tot*_ duty time (sec), *VA* alveolar ventilation (ml/min), *VD/VT* percentage of dead space (%), *Dead space ventilation* (ml/min), *Dead space washout* (ml/min), *Clearance half-time in trachea* (sec), *Comfort* respiratory comfort level measured by a scale, *PEIP* positive end-inspiratory pressure, *MIP* mean inspiratory pressure (cmH_2_O); *NA* not available

^a^Values are calculated

^b^Values extracted from graph using graph reader Website

^c^Values are reported as % of change from baseline

#### Airway pressure

**Fig. 5:**
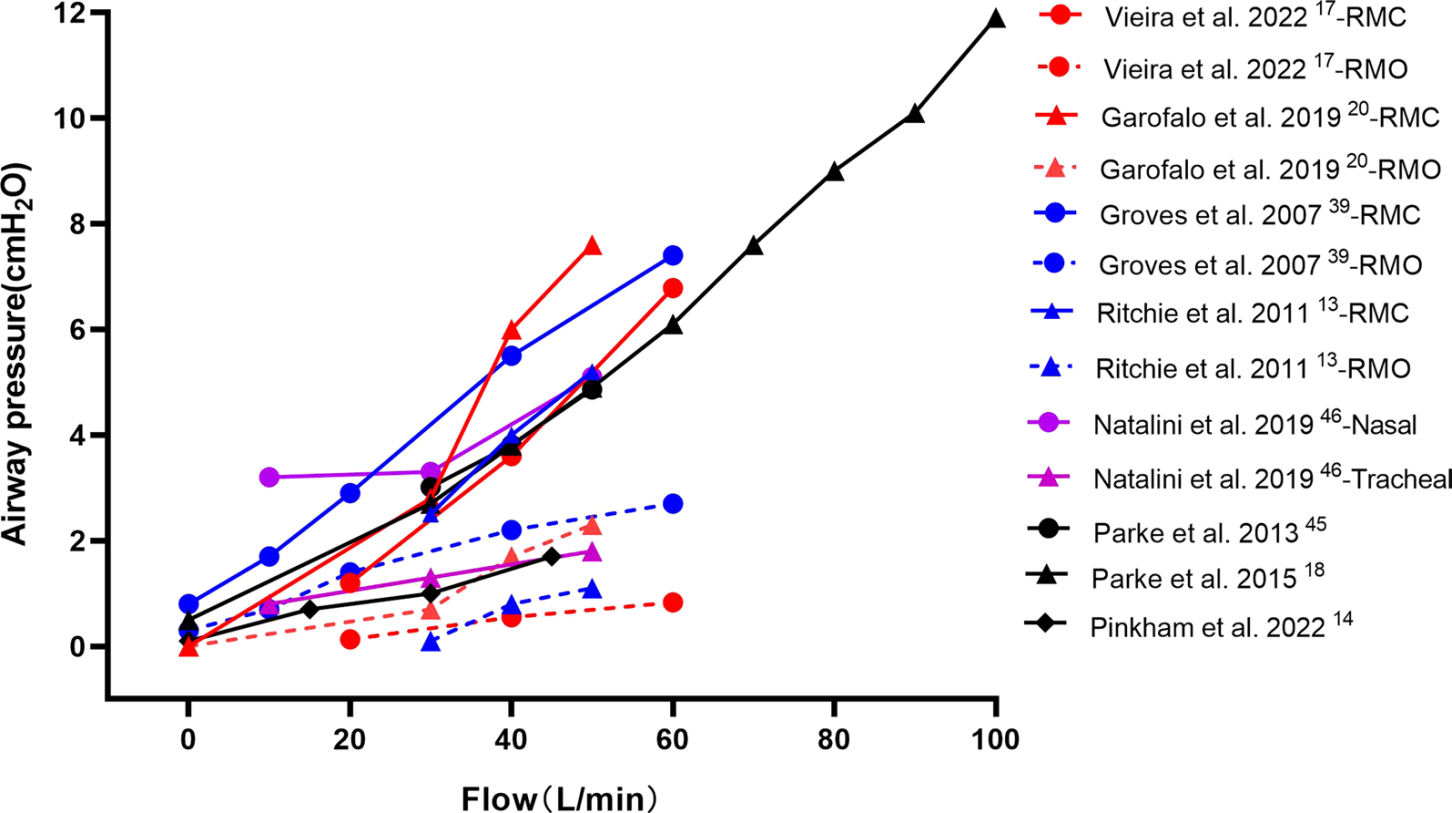
The relationship between airway pressures and HFNC flow settings. HFNC, high-flow nasal cannula

The flow-dependent PEEP effect from the in vitro studies has also been confirmed in healthy individuals, as end-expiratory esophageal pressures or hypopharyngeal pressure gradually increase when HFNC flows are increased (Fig. 5) [13, 16–18, 20, 39]. However, maintaining a constant PEEP with HFNC is challenging because it can significantly decrease with open-mouth breathing [13, 17, 20, 39]. When subjects opened their mouth, hypopharyngeal pressure dropped from 5.2 (3.5, 7.0) cmH_2_O to 1.1 (− 0.9, 2.4) cmH_2_O with HFNC set at 50 L/min [13], and nasopharyngeal pressure dropped from 6.8 to 0.8 cmH_2_O with HFNC set at 60 L/min [17]. Caution must be taken while using very high flows, such as 100 L/min, as it can provide nasopharyngeal pressure as high as 11.9 ± 2.7 cmH_2_O [18], and the tolerability is concerning.

#### End-expiratory lung impedance

Besides the potential for significantly elevated airway pressure, an uneven distribution of delivered gas across lung regions from different HFNC flows may also pose a risk for regional overdistension. Three studies evaluating ventilation distribution across lung regions from various HFNC flows using electrical impedance tomography (EIT) report that global end-expiratory lung impedance (EELI) increases as HFNC flow increases [17–19]. However, increases in EELI mainly occur in non-dependent regions of the lung. Plotnikow et al. reported an increase in EELI by 35% from baseline (room air) to HFNC set at 30 L/min and by 22% from 30 to 50 L/min in the non-dependent regions [19]. In the lung-dependent regions, the EELI only increased by 18% and 7.7%, respectively [19]. Since the non-dependent lung regions are most likely open normally, these findings suggest a potential risk of over-distending the non-dependent region, resulting in lung injury.

#### Swallow function

Three studies have investigated the effects of flows on swallow function among healthy volunteers during HFNC therapy [41–43]. Sanuki et al. [41] reported reduced latency time of the swallow reflex with HFNC flows being increased from 15 to 45 L/min when healthy volunteers swallowed 5 mL of distilled water over 3 s. Thus, they concluded that HFNC might enhance swallowing function [41]. However, Arizono et al. reported the opposite findings, as choking was observed when HFNC flows were ≥ 40 L/min in the 30 mL swallow test [42]. Additionally, they noted that swallowing efforts were greater with HFNC flows ≥ 20 L/min than 10 L/min [42]. Allen and Galek found a flow-dependent influence on the duration of laryngeal vestibule closure (dLVC) among their healthy volunteers [43]. Since LVC is a protective reflex that helps to prevent aspiration, the authors suggest that dLVC modulation from HFNC flows might help prevent aspiration [43]. Notably, a large variation of dLVC between HFNC flows of 50 and 60 L/min was found, underscoring the reality that further research on the impact of HFNC flows on swallow function is needed [43]. Oral feeding during HFNC therapy should be closely monitored, especially in severe hypoxemic patients who might need treatment escalation and those with dysphagia or at high risk for aspiration.

### Patients with acute hypoxemic respiratory failure

#### Oxygenation

During HFNC therapy for patients with AHRF, oxygenation has been assessed using SpO_2_/F_I_O_2_ (SF) ratio [6, 50], PaO_2_/F_I_O_2_ (PF) ratio [7, 8], and the ROX index (= SF/RR) [6, 9, 10]. Three studies have reported that oxygenation improves as HFNC flows increase (Table 2) [6–8], while Zhang et al. [9] found no significant changes in ROX index between room air and HFNC flow of 60 L/min in patients with mild hypoxemia. Likewise, Mauri et al. reported that 30% (17/57) of AHRF patients had an unchanged or decreased ROX index when HFNC flows were increased from 30 to 60 L/min. Their further analysis revealed that the 17 patients had a higher SF ratio and ROX index at 30 L/min, compared to the other 40 patients who presented an increase in ROX index with increasing flow [10]. Interestingly, the same authors implemented a study 2 years earlier on similar patient populations, and they found that 30% of patients had decreased PF ratios after increasing flows from 30 or 45 to 60 L/min [7].

**Table 2:** Physiological effects of flow settings during HFNC treatment for hypoxemic patients

Author/year	Study design	*N*	Patients	Position	Flow (L/min)	FiO_2_ or SpO_2_	RR (breaths/min)	$\Delta$ EELI_glob_ (ml)	PF or SF	ROX	PEEP (cmH_2_O)	PTP (cmH_2_O s/min)	Others
Mauri et al. 2017 [7]	Prospective	17	AHRF	Semi-recumbent position	12	SpO_2_ 90–96%	24 ± 8	Baseline	151 ± 60	NA	NA	254.3 (160.2, 359.5)	VT: 443 ± 302 MV:.1 ± 4.09 $\Delta$ Pes: 9.4 (6.8,12.2)
					30		20 ± 7	74 ± 174	177 ± 74	NA	NA	173.5 (126.4, 256.4)	VT: 437 ± 314 MV: 7.0 ± 2.8 $\Delta$ Pes: 7.9 (5.9,11.8)
					45		19 ± 7	115 ± 142	187 ± 67	NA	NA	168.9 (110.3, 217.2)	VT: 435 ± 307 MV: 7.0 ± 2.9 $\Delta$ Pes: 8.1 (5.7, 9.5)
					60		18 ± 7	230 ± 237	205 ± 61	NA	NA	151.4 (111.8, 195.6)	VT: 429 ± 301 MV: 6.9 ± 2.1 $\Delta$ Pes: 6.8 (5.1, 9.3)
Pinkham et al. 2022 [14]	Prospective	8	Decannulated from tracheostomy tube	Not specified	0	SpO_2_ 93–97%^c^	18 ± 5	NA	NA	NA	0.1 (0, 1.3)	NA	I:E: 0.75 ± 0.14 VT: 410 ± 170 MV: 6.8 ± 2.8
					15		16 ± 3	NA	NA	NA	0.7 (0.2, 1.1)	NA	I:E:0.7 ± 0.18 VT: 420 ± 170 MV: 6.2 ± 2.2
					30		14 ± 4	NA	NA	NA	1.0 (0.3, 3.0)	NA	I:E:0.6 ± 0.15 VT: 460 ± 220 MV: 6.4 ± 3.5
					45		14 ± 4	NA	NA	NA	1.7 (0.7, 7.1)	NA	I:E: 0.5 ± 0.17 VT: 450 ± 210 MV: 5.7 ± 1.7
Delorme et al. 2017 [24]	Randomized crossover study	7	AHRF	Not specified	5 ± 4 (baseline)	SpO_2_ 90–94%	NA	NA	NA	NA	NA	152 (113, 171)	WOB: 4.1 (3.0, 4.7) $\Delta$ Pes: 8.6 (5.3, 9.9)
					20		NA	NA	NA	NA	NA	127 (95, 139)	WOB: 3.2 (2.1, 5.6) $\Delta$ Pes: 7.7 (4.9, 9.8)
					40		NA	NA	NA	NA	NA	87 (74, 145)	WOB: 3.1 (2.5, 4.2) $\Delta$ Pes: 5.7 (4.0, 8.4)
					60		NA	NA	NA	NA	NA	65 (34, 99)	WOB: 1.9 (1.0, 2.6) $\Delta$ Pes: 2.1 (1.9, 4.5)
Zhang et al. 2020 [9]	Prospective	24	AHRF or high risk for AHRF after extubation	Semi-recumbent position	0	F_i_O_2_ 0.30	22 ± 6	Baseline	NA	22.4 ± 5.4	NA	NA	NA
					20		20 ± 4	7.2 (− 5.1, 18.7)^d^	NA	23.9 ± 5.2	NA	NA	NA
					40		20 ± 3	10.6 (− 3.4, 42)^d^	NA	23.5 ± 4.4	NA	NA	NA
					60		21 ± 5	26.3 (10.7,60.8)^d^	NA	22.2 ± 4.3	NA	NA	NA
Li et al. 2021 [6]	Prospective	49	AHRF	Not specified	Matching PTIF	F_i_O_2_ ≥ 0.40 and SpO_2_ of 90–97%	23 ± 7	NA	161 ± 51	7.5 ± 3.2	NA	NA	NA
					10 L/min above PTIF		23 ± 7	NA	181 ± 61	8.5 ± 4.0	NA	NA	NA
					20 L/min above PTIF		22 ± 6	NA	201 ± 67	9.8 ± 4.7	NA	NA	NA
					30 L/min above PTIF		23 ± 6	NA	207 ± 70	10.0 ± 4.7	NA	NA	NA
Basile et al. 2020 [8]	Prospective	12	AHRF	Semi-recumbent position	0.5 L/kg/min	SpO_2_ 90–96%	20 ± 6	Baseline	194 ± 96	NA	NA	NA	NA
					1.0 L/kg/min		17 ± 5	366.7 ± 1264^c^	211 ± 106	NA	NA	NA	NA
					1.5 L/kg/min		18 ± 6	760.8 ± 1096^c^	219 ± 118	NA	NA	NA	NA
Butt et al. 2021 [44]	Retrospective	19	Post-extubation	Not specified	20	SpO_2_ 92–97%	NA	NA	NA	NA	NA	NA	Comfort: 6.9 (6.2, 7.7)^ab^
					30		NA	NA	NA	NA	NA	NA	Comfort: 8.6 (8.0,9.0)^ab^
					40		NA	NA	NA	NA	NA	NA	Comfort: 8.58 (8.0, 9.0)^ab^
					50		NA	NA	NA	NA	NA	NA	Comfort: 6.8 (6.3, 7.2)^ab^
					60		NA	NA	NA	NA	NA	NA	Comfort: 3.0 (2.3, 3.7)^ab^
Parke et al. 2013 [45]	Prospective	15	Cardiac surgery	Not specified	30	Not specified	NA	NA	NA	NA	3.01 ± 1.18	NA	MAP: 1.5 ± 0.6 MPP:1.71 ± 0.73 MEP: 2.1 ± 0.83 MIP: 0.55 ± 0.38
					40		NA	NA	NA	NA	3.81 ± 1.45	NA	MAP 2.2 ± 0.8 MPP:2.48 ± 0.94 MEP: 2.88 ± 1.04 MIP: 1.11 ± 0.51
					50		NA	NA	NA	NA	4.86 ± 1.79	NA	MAP: 3.1 ± 1.2 MPP: 3.41 ± 1.24 MEP: 3.81 ± 1.33 MIP: 1.77 ± 0.69
Mauri et al. 2019 [10]	Retrospective	57	AHRF	Semi-recumbent position	30	SpO_2_ 90–96%	NA	437 ± 314	NA	10.2 (7.2, 13.3)	NA	NA	NA
					60		NA	429 ± 301	NA	11.1 (8.8, 13.9)	NA	NA	NA
Natalini et al. 2019 [46]	Prospective	5	Decannulated from tracheostomy tube	Not specified	10	SpO_2_ 92–98%	NA	NA	NA	NA	Nasal 3.2 (2.3, 5.9)^b^ Tracheal 0.8 (0.5, 1.2)^b^	NA	MEP nasal: 2.6 (1.6, 5.5)^b^ MEP tracheal: 0.5 (0.3, 0.7)^b^
					30		NA	NA	NA	NA	Nasal 3.3 (2.8, 5.5)^b^ Tracheal 1.3 (0.9,1.6)^b^	NA	MEP nasal: 2.6 (1.9, 4.2)^b^ MEP tracheal: 0.9 (0.6, 1.1) ^b^
					50		NA	NA	NA	NA	Nasal 5.1 (4.2, 7.7)^b^ Tracheal 1.8 (1.4,2.2)^b^	NA	MEP nasal: 3.9 (3.1, 6)^b^ MEP tracheal: 1.2 (1,1.5)^b^
Mauri et al. 2018 [50]	Prospective	40	AHRF	Semi-recumbent position	30 (Temp 31)	SpO_2_ 92–98%	22 (18, 29)	NA	238 (192, 246)	NA	NA	NA	Comfort: 3 (2, 5)
					30 (Temp 37)		23 (19, 29)	NA	240 (190, 248)	NA	NA	NA	Comfort: 4 (2, 5)
					60 (Temp 31)		22 (19, 25)	NA	240 (196, 248)	NA	NA	NA	Comfort: 3 (1, 5)
					30 (Temp 37)		23 (19, 26)	NA	240 (196, 248)	NA	NA	NA	Comfort: 3 (2,5)

*FiO*_*2*_ Fraction of inspired oxygen (%),*SpO* *SpO*_*2*_ Saturation of oxygenation using pulse oximeter (%), *RR* respiratory rate (breath/min), $\Delta$ *EELI*_*glob*_ global change in end-expiratory lung impedance measured by electrical impedance tomography, *PF* PaO_2_/F_i_O_2_, *SF* SpO_2_/F_i_O_2_, *ROX index* [(SpO_2_/F_i_O_2_)/RR], *PEEP* positive end-expiratory pressure measured by pharyngeal or tracheal catheter, *PTP* esophageal pressure-time product per minute (cmH_2_O·s/min), *VT* tidal volume (ml), *MV* minute ventilation (L/min), $\Delta$ *Pes* esophageal pressure swings (cmH_2_O), *I:E* inspiratory to expiratory ratio, *Comfort* respiratory comfort level measured by a scale, *MPP* mean plateau pressure (cmH_2_O), *MEP* mean expiratory pressure (cmH_2_O), *MIP* mean inspiratory pressure (cmH_2_O), *MAP* mean airway pressure (cmH_2_O), *Temp* temperature (°C), *AHRF* acute hypoxemic respiratory failure, *PTIF* peak tidal inspiratory flow, *NA* not available

^a^Values reported as 95% CI

^b^Values extracted from graph using graph reader Website

^c^Values reported directly from the author

^d^Values are reported as % of change from baseline

#### End-expiratory lung impedance

Similar to the findings in healthy individuals, increasing flows also improves global EELI in patients with AHRF (Additional file 1: Table S3, appendix p5) [7–9]. Increasing HFNC flow generates a greater end-expiratory lung volume and PEEP [45, 46], which may cause recruitment that mainly occurs in dependent lung regions. However, it may also generate overdistension that is more pronounced in non-dependent lung regions. It appears that changes in oxygenation, that correlate with changes in EELI [9], depend on the balance of alveolar recruitment and overdistension.

The regional distribution of the aeration depends on HFNC flows and patients [7–9]. When flows were increased from 30 to 60 L/min, Mauri et al [7] reported that EELI increased, but not by a significant amount. Interestingly, when compared to EELI with a facemask, EELI in dependent lung regions significantly increased with HFNC at 60 L/min, while EELI in non-dependent regions remained stable. These findings suggest more recruitment in dependent lung regions than overdistension in non-dependent regions [7]. In a follow-up study that included 12 patients with AHRF [8], the same group of authors compared the effect of different flows that were set based on the patient’s predicted body weight (0.5, 1.0, and 1.5 L/Kg/min). They utilized median flows of 35, 65, and 100 L/min, respectively [8]. Compared to EELI at 0.5 L/Kg/min, EELI in non-dependent lung regions increased at 1.0 L/Kg/min and 1.5 L/Kg/min (*p* = 0.01), with significance reached at 1.5 L/Kg/min (*p* < 0.05), while EELI in dependent lung regions remained constant (*p* = 0.548). Both studies suggest that HFNC flows at 60–65 L/min may cause more recruitment than overdistention, while high flows (such as 100 L/min) may result in lung overdistention, especially in non-dependent lung regions [8]. The large variability between patients in these two studies should be noted, suggesting that personalized flow titration based on its physiological effects may be a pragmatic approach to be used at the bedside. For example, Mauri et al. [7] reported that 37% of patients had improvement in EELI in dependent regions with HFNC at 30 or 45 L/min, but not at 60 L/min. Similarly, Zhang et al. [9] compared EELI at baseline (room air) versus 60 L/min and used the regional recruitment (recruited pixels) to define the potential of lung recruitment, in which recruited pixels > 10% pixels at 60 L/min than at baseline was defined as the high potential of recruitment. They found that 13 in 24 patients (54%) had a high potential for recruitment. For these patients, they noted that recruitment mainly occurred in dependent lung regions when HFNC flow was increased from 0 to 60 L/min [9]. For the rest of the patients included in the study, seven had unchanged EELI and four had overdistension without lung recruitment, occurring mainly in the non-dependent lung regions [9]. The difference in regional volume distribution from various flows in the three studies might be due to the factors that cause different responses to PEEP, including disease severity, etiology, duration of pulmonary disease, and closed- vs open-mouth breathing. Regardless, close monitoring of individuals' responses in regional lung volumes to different flows might help avoid overdistension and lung injury.

#### Inspiratory efforts

**Fig. 6:**
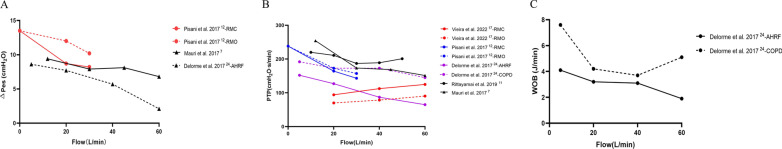
Effects of flow settings on $\Delta$ Pes (**A**), PTP (**B**), and WOB (**C**). HFNC, high-flow nasal cannula; $\Delta$ Pes: esophageal pressure swings (cmH_2_O); PTP: esophageal pressure–time product per minute (cmH_2_O·s/min); WOB, work of breathing (J/min)

Beyond the regional distribution of volume, dynamic transpulmonary pressure reflects the patient inspiratory effort and lung stress, which is associated with lung injury. Changes in esophageal pressure ($\Delta$Pes) are a surrogate for inspiratory effort [55]. When HFNC flows were increased, inspiratory effort (Fig. 6A), pressure–time product (Fig. 6B), and WOB (Fig. 6C) decreased [7, 24]. Mauri et al. described an exponential decay correlation between HFNC flows and patient inspiratory effort [7]. The reduction in the patient effort was caused by several factors, such as recruitment of atelectatic regions, an increase in dead space washout, a decrease in nasal resistance, an improvement in secretion clearance, and an increase in dynamic lung compliance [8, 24]. However, they also found that 43% of patients had increased $\Delta$Pes when HFNC flows were increased from 30 or 45 to 60 L/min [7]. The patients that demonstrated an increase in $\Delta$Pes might have had a compliance decrease due to alveolar overdistention, particularly in the previously relatively well-aerated regions of their lungs with 30 L/min [7]. Thus, due to the concerns that lung injury might occur in patients who have no recruitment with increasing HFNC flows, it has been suggested to titrate flow based on the inspiratory effort [9].

#### Dead space and respiratory rates

Another important flow-dependent effect is the reduction in dead space. Pinkham et al. [14] reported that exhaled gas rebreathing volume increased as RR increased [14]. Moreover, when RR was ≥ 25 breaths/min, rebreathing volume with HFNC at 20 L/min was greater than when flows were 40 and 60 L/min. However, there were no differences in rebreathing volume among the three flows when RR was 15 breaths/min. Thus, the authors proposed that RR could be used as an indicator that HFNC flows should increase when RR is high [14]. Five studies to date have shown an overall reduction in RR when HFNC flows are increased for patients with AHRF [6–9, 14], and two of them reported significant reductions in minute ventilation [7, 14].

#### Patient comfort

Mauri et al. [50] compared 30 vs 60 L/min and 31 °C vs 37 °C for 40 patients with AHRF. Patient comfort was lower at 37 °C than at 31 °C, but comfort was not different between flows set at 30 and 60 L/min. Despite large interindividual variability, they reported a higher comfort score with the lowest temperature and the highest flow in the subgroup of patients whose F_I_O_2_ was ≥ 0.45 [50]. However, this does not necessarily mean that the optimal temperature to achieve better comfort should be 31 °C. As temperature differences were only assessed by comparing 20-min periods, tolerance may improve over time. Similarly, three other studies evaluating comfort in patients with AHRF reported no significant differences between flows set below 60–65 L/min [6, 8, 44]. Patient comfort was significantly lower when HFNC flows were set at ~ 100 L/min [8]. Interestingly, Butt et al. [44] found that patient comfort significantly decreased when HFNC flow was set at ≥ 50 L/min among post-extubation patients. Thus, for patients with AHRF who are more hypoxemic and present higher inspiratory demands, higher flows may be associated with better comfort. Once again, variability in patient response to different flows and temperatures highlights the importance of personalization of HFNC settings.

#### Treatment failure

Only one study compared the effect on clinical outcomes of using different flows, which was conducted among cardiac surgery patients with post-extubation hypoxemia with HFNC at 40 versus 60 L/min. The authors reported clinically meaningful differences in treatment failure rate between the two groups (30.3% vs 12.1%, *p* = 0.11), with reintubation rates of 15.2% and 6.1%, respectively [27].

#### Individualization of HFNC flow settings

For patients with AHRF, it appears that higher flows improve oxygenation and lung compliance, and reduce WOB. These clinical benefits are, at least in part, due to the increase in PEEP and end-expiratory lung volume. However, it should be noted that not all patients respond to higher flows, and some patients might have uneven volume distribution in their lungs which might lead to alveolar overdistention in non-dependent lung regions. To individualize flow settings based on patient needs (PTIF), Li et al. [6] set HFNC flow to match the PTIF or 10, 20, and 30 L/min above PTIF. They found that the SF ratio and ROX index increased as flow increased. However, the ROX index plateaued when the flow was set at 1.67 times PTIF [6]. Currently, there is no commercially available device to monitor PTIF breath-by-breath, so this approach may not be feasible. Butt et al. measured PTIF during mechanical ventilation before extubation and set HFNC flows based on the patient’s comfort after extubation which ranged between 30 and 50 L/min [44]. They reported a significant correlation between PTIF and HFNC flows [44]. Thus, PTIF measured on a mechanical ventilator before extubation might be a reference for flow settings during post-extubation HFNC treatment. However, more studies are needed to validate the accuracy of these methods and explore the effects of these methods on regional lung aeration and inspiratory efforts. Additionally, the clinical benefits, such as the need for reintubation, of applying this method to set flows during HFNC treatment are unknown.

Before the aforementioned methods are clearly demonstrated and devices are commercially available, clinicians may use pragmatic assessments that can be easily measured at the bedside, such as SF ratio, RR, ROX index, and comfort to titrate HFNC flows. One possible strategy could be that when oxygenation starts to plateau, clinicians might stop the upward flow titration and return to the previous flow when the plateau is first recognized. Patient comfort is also a key consideration during HFNC flow titration. However, it should be noted that the changes in oxygenation, RR, and comfort to HFNC flows may not be sensitive in patients with mild hypoxemia.

### Patients with COPD

The main effects of HFNC flows for COPD are improved ventilation efficiency [11, 12, 21–24], pressure–time product (Fig. 6B), [11, 12] WOB (Fig. 6C), [24] and comfort [12, 21, 22]. When HFNC flows were increased, seven studies reported reductions in RR [11, 12, 21, 22], and two studies reported increases in *Vt *(Table 3) [12, 21]. Additionally, longer exhalation times were observed [12], which might help alleviate air-trapping and WOB. With increased flows, minute ventilation was the same or lower [21], but PaCO_2_ or PtCO_2_ was lower [11, 12, 21–24]. This interesting finding implies improved alveolar ventilation.

**Table 3:** Physiological effects of flow settings during HFNC treatment for patients with COPD

Author/year	Study design	*N*	Patients	Activity/Breathing pattern	Flow (L/min)	FiO_2_ or SpO_2_	RR (breaths/min)	PaCO_2_ or PtCO_2_	VT (ml)	MV (L/min)	PTP (cmH_2_O x Sec/min)	Others
Bräunlich et al. 2016 [21]	Prospective	54	Stable COPD gold C or D	Not specified	20	Not specified	11.6 ± 3.6	91 ± 6.7	534 ± 215	6.0 ± 2.8	NA	MAP: 0.92 ± 0.5 RSBI: 27.4 ± 17.9
					30		11.1 ± 3.6	87.4 ± 6.2	524 ± 228	5.6 ± 2.6	NA	MAP: 1.44 ± 0.8 RSBI: 28 ± 18.9
					40		10.3 ± 3.3	NA	562 ± 249	5.5 ± 2.5	NA	MAP: 2.14 ± 1 RSBI: 25.1 ± 19.9
					50		9.9 ± 2.7	NA	559 ± 260	5.4 ± 2.5	NA	MAP: 3.01 ± 1 RSBI:25.4 ± 18.3
Pisani et al. 2017 [12]	Randomized controlled trial	14	COPD with CHRF	MC	0	Not specified	24.8 ± 2.3	61.2 ± 9.2	314 ± 84	NA	238.3 ± 82.1	PEEPi, dyn: 2.12 ± 0.9 Comfort: 7 (5,8) $\Delta$ Pes: 13.5 ± 6.7
				MC	20		19.0 ± 5.2	57.2 ± 11.7	391 ± 106	NA	164.2 ± 51.3	PEEPi, dyn: 1.48 ± 0.7 Comfort:5.5 (5,8) $\Delta$ Pes: 8.7 ± 4.1
				MO			20.8 ± 5.8	NA	NA	NA	172.7 ± 45.4	$\Delta$ Pes: 12 ± 5.8
				MC	30		18.7 ± 3.6	55.7 ± 10.6	364.2 ± 66	NA	143.2 ± 48.9	PEEPi, dyn: 1.03 ± 0.6 Comfort: 5.5 (2,8) $\Delta$ Pes: 8.2 ± 3.7
				MO			19.6 ± 2.8	NA	NA	NA	157.3 ± 56.9	$\Delta$ Pes: 10.2 ± 5.2
Bräunlich et al. 2018 [23]	Prospective	36	Stable COPD with CHRF	Not specified	20	Not specified	NA	94.2 ± 8.3^a^	NA	NA	NA	PaO_2_: 99.2 ± 18.9^a^ MAP: 0.57 ± 3.8
					40		NA	93.5 ± 4.4^a^	NA	NA	NA	PaO_2_: 93.1 ± 15.7^a^ MAP: 2.3 ± 1.6
Mckinstry et al. 2018 [22]	Randomized controlled trial	48	Stable COPD	Not specified	0	F_I_O_2_ 0.21	17.5 ± 4.8	38.8 ± 5^b^	NA	NA	NA	NA
					15		16.0 ± 5.7	38.0 ± 5.3^b^	NA	NA	NA	Comfort:11 (4.5,17,4)
					30		13.4 ± 5.2	37.3 ± 6.0^b^	NA	NA	NA	Comfort:22 (13.8,26.7)
					45		13.3 ± 4.8	36.3 ± 5.6^b^	NA	NA	NA	NA
Rittayamai et al. 2019 [11]	Prospective	12	Hypercapnic COPD	Not specified	10	SpO_2_ ≥ 92%, F_I_O_2_ 0.30–0.40	24 (20,29)	41 ± 7^b^	NA	NA	220 ± 100	SF:271 ± 3^c^
					20		23 (18,28)	41 ± 8^b^	NA	NA	211 ± 90	SF:274 ± 3^c^
					30		21 (18,27)	41 ± 7^b^	NA	NA	187 ± 84	SF:277 ± 3^c^
					40		21 (18,27)	41 ± 7^b^	NA	NA	189 ± 87	SF:277 ± 2^c^
					50		21 (18,26)	41 ± 7^b^	NA	NA	201 ± 86	SF: 280 ± 2^c^
Delorme et al. 2017 [24]	Randomized crossover trial	5	Hypercapnic	Not specified	5 ± 4 (baseline)	SpO_2_ 90–94%	NA	NA	NA	15.3 (11.2, 20.0)	192 (161, 245)	WOB: 7.6 (4.0, 8.4)
					20		NA	NA	NA	12.1 (7.4, 15.0)	173 (105, 252)	WOB: 4.2 (2.4, 5.2)
					40		NA	NA	NA	9.6 (7.6, 20.4)	173 (147, 228)	WOB: 3.7 (3.0, 6.7)
					60		NA	NA	NA	12.2 (6.6, 16.3)	145 (79, 235)	WOB: 5.1 (1.8, 5.4)

*FiO*_*2*_ fraction of inspired oxygen (%), *SpO*_*2*_ saturation of oxygenation using pulse oximeter (%), *PaCO*_*2*_ arterial partial pressure of carbon dioxide (mmHg), *PtCO*_*2*_ transcutaneous partial pressure of carbon dioxide (mmHg), *PcCO*_*2*_ capillary partial pressure of carbon dioxide (mmHg), *RR* respiratory rate (breath/min), $\Delta$* EELI*_*glob*_ global change in end-expiratory lung impedance measured by electrical impedance tomography, *PF* PaO_2_/F_i_O_2_, *SF* SpO_2_/F_i_O_2_, *ROX index* [ (SpO_2_/F_i_O_2_)/RR], *PEEP* positive end-expiratory pressure measure by pharyngeal or tracheal catheter, *PTP* esophageal pressure-time product per minute (cmH_2_O·s/min), *VT* tidal volume (ml), *MV* minute ventilation (L/min), $\Delta$ Pes esophageal pressure swings (cmH_2_O), *MAP* mean airway pressure (mbar), *PEEPi* dyn dynamic intrinsic positive end-expiratory pressure (cmH_2_O), *WOB* work of breathing (J/min), *Comfort* respiratory comfort level measured by a scale, *COPD* chronic obstructive pulmonary disease, *CHRF* chromic hypercapnic respiratory failure, *MO* mouth opened, *MC* mouth closed, *RSBI* rapid shallow breathing index, *HFNC* high-flow nasal cannula, *NA* not available

^a^Value reported as change from baseline (%)

^b^Value reported as PtCO_2_

^c^Values are calculated

### Patients during procedural sedation

Three RCTs compared the effectiveness of different flows during procedural sedation (Additional file 1: Table S4, appendix p6) [47–49], with two RCTs comparing HFNC at 40 vs 60 L/min [47, 48] and one comparing HFNC at 30 vs 50 L/min [49]. Compared to HFNC at low flows (30 or 40 L/min), HFNC at higher flows (50 or 60 L/min) had greater oxygenation at the end of the procedure (Additional file 1: Fig. S2, appendix p7) [47–49] and required fewer interventions, such as jaw lifting, during the procedure [49].

## Conclusion

The physiological effects of HFNC oxygen therapy are flow-dependent and are maximized when the flow exceeds PTIF. However, PTIF varies between patients and disease conditions. Higher flows result in improved oxygenation and dead space washout and can reduce work of breathing. Notably, higher flows can also lead to alveolar overdistention in non-dependent lung regions and to patient discomfort. The impact of flows on different patients is largely heterogeneous. Individualizing flow settings during HFNC treatment is necessary, and titrating flow based on clinical findings like oxygenation, RR, and patient comfort is a pragmatic way forward, at least for now.

## Supplementary Information

**Additional file 1**. Assessments of risk of bias for included trials (**Figure S1** and **Table S1**); report of peak tidal inspiratory flow (**Table S2**); global and regional end-expiratory lung volume with different HFNC flows (**Table S3**); RCTs (**Table S4**) and gas exchanges (**Figure S2**) of different flows during procedural sedation. 

## Data Availability

Data will be available 36 months after article publication to researchers who provide a methodologically sound and ethically approved proposal, for any purpose of analysis.
