# Supplementary material for: The effects of flow settings during high-flow nasal cannula support for adult subjects: a systematic review
Source: Crit Care. 2023 Feb 28;27:78. doi: 10.1186/s13054-023-04361-5 (PMC9974062; doi:10.1186/s13054-023-04361-5)

The effects of flow settings during high-flow nasal cannula support: A Systematic Review

Supplementary file

Jie Li,[1#*] PhD, RRT; Fai AlBuainain,[1,2#] BS; Wei Tan,[3] PhD; J. Brady Scott,[1] PhD, RRT; Oriol Roca,[4,5,6] MD, PhD; Tommaso Mauri,[7,8] MD, PhD.

**Figure S1. Assessments of risk of bias for randomized controlled or crossover trials**	**p 2**
**Table S1: Assessment on risk of bias for included non-randomized trials using Newcastle-Ottawa Scale**	**p 3**
**Table S2: Report of peak tidal inspiratory flow for adults**	**p 4**
**Table S3. Analysis of global and regional end-expiratory lung volume with different HFNC flow**	**p 5**
**Table S4. RCTs of different flows during procedural sedation**	**p 6**
**Figure S2. Gas exchanges during procedural sedation with different flows**	**p 7**

Figure S1: Assessments of risk of bias for randomized controlled or crossover trials

Unique ID	Study ID	Experimental	Comparator	D1	D2	D3	D4	D5	Overall
Mauri 2017	NA	30,45,60 L/min	face mask 12L/min	!	+	+	+	!	!
Basile 2020	NA	1.0 and 1.5 L/kg/min	0.5 L/kg/min	!	+	+	+	!	!
Delrome 2017	NCT02494154	40 and 60 L/min	20 L/min	!	+	+	+	+	!
Natalini 2019	NA	10, 30, 50 L/min	face mask 8 L/min	!	+	+	+	!	!
Parke 2013	ACTRN 12609000305224	40 and 50 L/min	30 L/min	!	+	+	+	+	!
Mauri 2019	NA	60 L/min	30 L/min	!	+	+	+	!	!
Theologou 2021	NCT03282552	40 and 60 L/min	venturi mask 15 L/min	+	!	+	!	+	!
Braunlich 2018	NCT02504814	40 L/min through both pro	20 L/min through both prongs	!	+	+	+	+	!
Mckinstry 2017	ACTRN12615000471583	15, 30 and 45 L/min	0 L/min	+	+	+	+	+	+
Pisani 2017	NCT02363920	HFNC of 20 and 30 L/min , I	Nasal cannula	!	+	+	+	+	!
Allen 2021	NA	10, 20, 30,40, 50 and 60 L/ı	0 L/min	!	+	+	+	!	!
Arizono 2021	UMIN 000041825	10, 20, 30, 40, 50 and 60 L/	0 L/min	!	+	+	+	+	!
Sanuki 2017	NA	15, 30 and 45 L/min	0 L/min	!	+	+	+	!	!
Delrome 2020	NCT02495675	20,40 and 60 L/min	Nasal cannula 5 L/min	+	+	+	+	+	+
Moller 2017	NCT01509703	30 and 45 L/min	15 L/min	!	+	+	+	+	!
Le Moigne 2021	NCT04096183	30 and 60 with 2 mouth coı	0 L/min	!	+	+	+	+	!
Vieira 2022	NCT03902639	20, 40 and 60 L/min with 2	CPAP 4 cmH2O	!	+	+	+	+	!
Ritchie 2011	NA	20, 30, 40, 50 and 60 L/min	10 L/min	+	+	+	+	!	!
Yi 2020	CHiCTR1800016621	40 and 60 L/min	nasopharyngeal airway with 0 L/min	!	+	+	+	+	!
Lucangelo 2012	NA	40 and 60 L/min	venturi mask 40L/min	+	+	+	+	!	!
Sago 2015	NA	30 and 50 L/min	Nasal cannula 5 L/min	!	+	+	+	!	!
Mauri 2018	NA	60 L/min (31 and 37 °C)	30 (31 and 37 °C)	!	+	+	!	!	!

+ Low risk

! Some concerns

- High risk

D1 Randomisation process

D2 Deviations from the intended interventions

D3 Missing outcome data

D4 Measurement of the outcome

D5 Selection of the reported result

Table S1: Assessment on risk of bias for included non-randomized trials using Newcastle-Ottawa Scale.

Author, year	Selection				Comparability		Outcome			Quality#
	Representativeness of the exposed cohort	Selection of the non-exposed cohort	Ascertainment of exposure	Outcome of interest not present at start of study	Controls for age, sex, and marital status	Controls of other factors	Assessment of outcome	Follow-up long enough for outcomes to occur	Adequacy of follow-up of cohorts	
Li, 2021	*	*	*	MD		*	*	*	*	Good
Butt, 2020		*	*				*	*	*	Poor
Pinkham			*	*			*		*	Poor
Zhang, 2020	*	*	*	*		*	*	*	*	Good
Braunlich, 2016		*	*	MD		*	*	MD	*	Fair
Rittayami, 2019		*	*	MD		*	*	*	*	Fair
Garofalo, 2019	*	*	*	*		*	*	*	*	Good
Okuda, 2017		*	*	*		*	*		*	Good
Plotnikow, 2018		*	*	*		*	*		*	Good
Groves 2007		*	*	*	*	*	*	MD	*	Good
Parke 2015		*	*	*		*	*		*	Good

MD, missing data. #The quality (good, fair, and poor) was defined based on the following criteria: Good - 3 or 4 stars in 'Selection' domain AND 1or 2 stars in 'Comparability' domain AND 2 or 3 stars in 'Outcome' domain; Fair - 2 stars in 'Selection' domain AND 1or 2 stars in 'Comparability' domain AND 2 or 3 stars in 'Outcome' domain; Poor - 0 or 1 star in 'Selection' domain OR 0 star in 'Comparability' domain OR 0 or 1 star in 'Outcome' domain.

Table S2: Report of peak tidal inspiratory flow for adults

Author /year	Study design	N	Population	Evaluation method	Activity/breathing pattern	PTIF (L/min)	MIF (L/min)
Butt et al. 2021[44]	Prospective	19	Mechanically ventilated	Ventilator (Drager V500)	NA	60 (40, 80)[* ab]	40 (25, 55) [*ab]
Li et al, 2021[6]	Prospective	49	AHRF	NICO2 flow sensor with mask	Rest	34 ± 9; 31 (27, 42)	NA
Chanques et al, 2013 [54]	Prospective	10	Post tracheostomy	Minitrach™ II cannula	Rest	30 (27, 32)	NA
Li et al, 2021[53]	RCT	75	Asthma/COPD	Spirometer	Rest	36.4 (34.5, 38.2)	NA
Ritchie et al. 2011[13]	Prospective	10	Healthy volunteers	Spiroson-AS ultrasonic flow sensor	Rest	27.9 ± 9.2	NA
					Exercise	119.9± 20.0	NA
Anderson et al, 2006 [52]	Prospective	13	Healthy volunteers	Pneumotachometer	Light exercise	125.6 ± 29.2	100.3±18.9
					Moderate exercise	185.3±37.1	150.1±35.4
					Heavy exercise	254.7±57.8	218.4±53.7

Abbervations: **PTIF**: peak tidal inspiratory flow (L/min), **MIF**: mean inspiratory flow (L/min), **AHRF**: acute hypoxemic respiratory, **COPD**: chronic obstructive pulmonary disease, **PS**: pressure support, **PEEP**: positive end expiratory pressure

*Measured with endotracheal tube during invasive ventilation (PS 0, PEEP 0)

a Values extracted from graph using graph reader website

b Values are calculated

Table S3. Analysis of global and regional end-expiratory lung volume with different HFNC flow

	Author/year	Position	Flow (L/min)	$\Delta EELI_{global}$	$\Delta EELI_{non\text{-}dependent}$		$\Delta EELI_{dependent}$	
					ROI 1	ROI 2	ROI 3	ROI 4
AHRF	Mauri et al. 2017[7]	Semi-recumbent position	12 (facial mask)	baseline	baseline		baseline	
			30	74±174 ml	53±183 ml		31±119 ml	
			45	115±142 ml	64±133 ml		59±121 ml	
			60	230±237 ml [a]	128±185 ml		93±150 ml [a]	
	Basile et al. 2020[8]	Semi-recumbent position	0.5 L/kg/min (35)	baseline	baseline		baseline	
			1.0 L/kg/min (65)	366.7 ±1264 au [a,d]	513.9±1239.1 au [d]		-147.2±1000 au [d]	
			1.5 L/kg/min (100)	760.8 ±1096 au [a,d]	819±1099 au [a,d]		-58.2±953 au [d]	
	Zhang et al. 2020[9]	Semi-recumbent position	0	baseline	baseline	baseline	baseline	baseline
			20	7.2 (-5.1, 18.7)%	0.3(-0.1,2.7)%	2.12 (-3.2,7.4)%	2.51 (-3.8, 6.2)%	0.21(0,0.4)%
			40	10.6 (-3.4, 42.0)% [a,b]	0.18 (0, 3.1)%	8.6 (-1.4,18.2)% [a,b]	3.94(-3.9, 14.5)%	0(0,1.8)%
			60	26.3(10.7,60.8)% [a,b,c]	1.61 (0,4.6)%	16.15(4.0,27.9)% [a,b]	14.3(4.0,32.3)% [a,b,c]	0.33(0,2.4)%
Healthy	Plotnikow et al. 2018 [19 f]	Supine	0	baseline	baseline		baseline	
		Setting 45°		1.05 (0.72, 1.34) Units [a]	0.2 (0.11, 0.34) Units [a]		0.33 (0.23, 0.47) Units [a]	
			30	1.12 (0.8, 2.01) Units [a,b]	0.27 (0.08, 0.45) Units [a,b]		0.39 (0.24, 0.64) Units [a,b]	
			50	1.44 (1.05, 2.16) Units [a,b,c]	0.33 (0.18, 053) Units [a,b,c]		0.42 (0.31, 0.65) Units [a,b,c]	

Abbervations: **AHRF**, acute hypoxemic respiratory failure; **EELI**, end expiratory lung impedanc**e; ROI,** region of interest

a vs. baseline, $p < 0.05$

b vs. 1st setting, $p < 0.05$

c vs. 2nd setting, $p < 0.05$

d Values reported directly from the author

e EELI results reported as au not ml in this study

f EELI results reported as unit not ml in this study

Table S4. RCTs of different flows during procedural sedation

Author/year	Study design	N	Patients	Flow (L/min)	FiO_2 or SpO_2	$PaCO_2$ (mmHg)	PF (%)	SpO_2 (%)	PaO_2 (mmHg)	others
Yi et al. 2020 [47]	RCT	23	Craniotomy with IVS	6	0.60	43.6±5.9	275.1±92.8	99.9±0.2	NA	**UAWO:** 10 **AWI:** 6 **Awakening duration:** 99 (85,113) min **Requiring treatment for increased BRS:** 6
		22		40		44.1±2.8	475±106.1	99.5±0.8	NA	**UAWO:** 3 **AWI:** 0 **Awakening duration:** 105 (75,136) min **Requiring treatment for increased BRS:** 3
		20		60		42.3±4.9	465.4±78	99.8±0.3	NA	**UAWO:** 1 **AWI:** 0 **Awakening duration:** 141.5 (98,198.5) min **Requiring treatment for increased BRS:** 5
Sago et al. 2015 [49]	RCT	10	Dental procedure with IVS	5	0.40	45.3±1.2 [a]	NA	95 (94,96) [a]	91±3.5 [a]	**Intervention**: 4 [a]
		10		30		44.3±1.9 [a]	NA	98 (97,99) [a]	178±9 [a]	**Intervention:** 2 [a]
		10		50		41.6±1.3[a]	NA	99 (98,100) [a]	225±11 [a]	**Intervention:** 0 [a]
Lucangelo et al. 2012 [48]	RCT	15	Bronchoscopy with pulmonary disease with IVS	40 VM	0.50	42.7 (41, 44.4)	165 (127.4, 199.2)	94 (92,96)	82.5 (63.7, 99.6)	NA
		15		40		43.2 (37.9, 47.6)	140.6(125.6, 153.6)	92 (90, 95)	70 (62.8, 76.8)	NA
		15		60		43.6 (42.4, 48)	244.8 (181.6, 366.8)	98 (97,99)	122.4 (90.8, 183.4)	NA

Abbervations: **FiO2:** fraction of inspired oxygen (%), **SpO2:** saturation of oxygen using pulse oximeter (%), **$PaCO_2$:** arterial partial pressure of carbon dioxide (mmHg), **PF:** PaO_2/ F_IO_2, **NPA**: nasopharyngeal airway, **UAWO**: upper airway obstruction (%), **AWI**: airway injury (%), **BRS**: brain relaxation score (%), IVS: intravenous sedation, **Interventions**: the procedures needed to improve ventilation such as jaw lifting or sniffing position. **RCT:** randomized control trial.
A Values extracted from graph using graph reader website

Figure S2. Gas exchanges during procedural sedation with different flows

A. $PaCO_2$

Study or Subgroup	Higher flow Mean	SD	Total	Lower flow Mean	SD	Total	Weight	Mean Difference IV, Random, 95% CI
Lucangelo, 2012	44.7	4.6	15	42.9	7.9	15	13.5%	1.80 [-2.83, 6.43]
Sago, 2015	41.6	1.3	10	44.3	1.9	10	53.3%	-2.70 [-4.13, -1.27]
Yi, 2020	42.3	4.9	20	44.1	2.8	22	33.3%	-1.80 [-4.25, 0.65]
**Total (95% CI)**			**45**			**47**	**100.0%**	**-1.79 [-3.66, 0.07]**

Heterogeneity: $Tau^2 = 1.18$; $Chi^2 = 3.45$, $df = 2$ ($P = 0.18$); $I^2 = 42\%$
Test for overall effect: $Z = 1.88$ ($P = 0.06$)

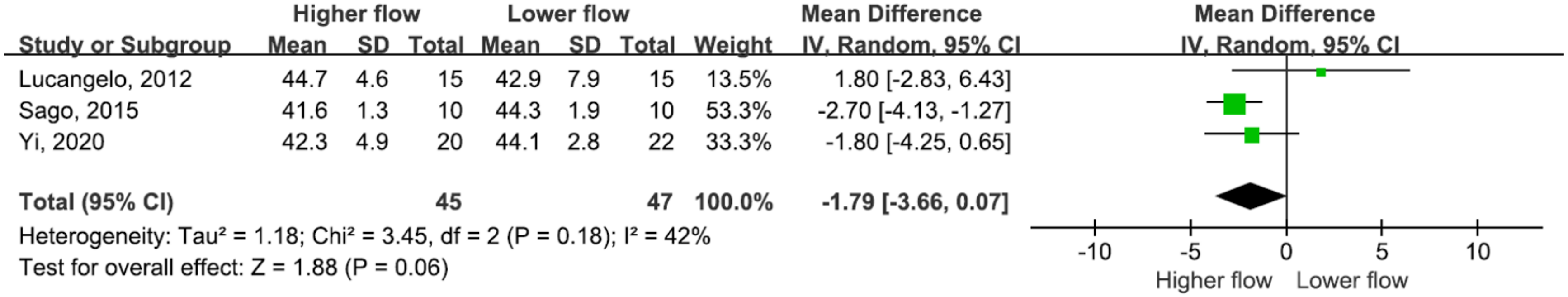

B. SpO_2

Study or Subgroup	Higher flow Mean	SD	Total	Lower flow Mean	SD	Total	Weight	Mean Difference IV, Random, 95% CI
Lucangelo, 2012	98	1.6	15	92.3	4.1	15	29.2%	5.70 [3.47, 7.93]
Sago, 2015	99	1.7	10	98	1.7	10	33.4%	1.00 [-0.49, 2.49]
Yi, 2020	99.8	0.3	20	99.5	0.8	22	37.5%	0.30 [-0.06, 0.66]
**Total (95% CI)**			**45**			**47**	**100.0%**	**2.11 [-0.41, 4.62]**

Heterogeneity: $Tau^2 = 4.36$; $Chi^2 = 22.53$, $df = 2$ ($P < 0.0001$); $I^2 = 91\%$
Test for overall effect: $Z = 1.64$ ($P = 0.10$)

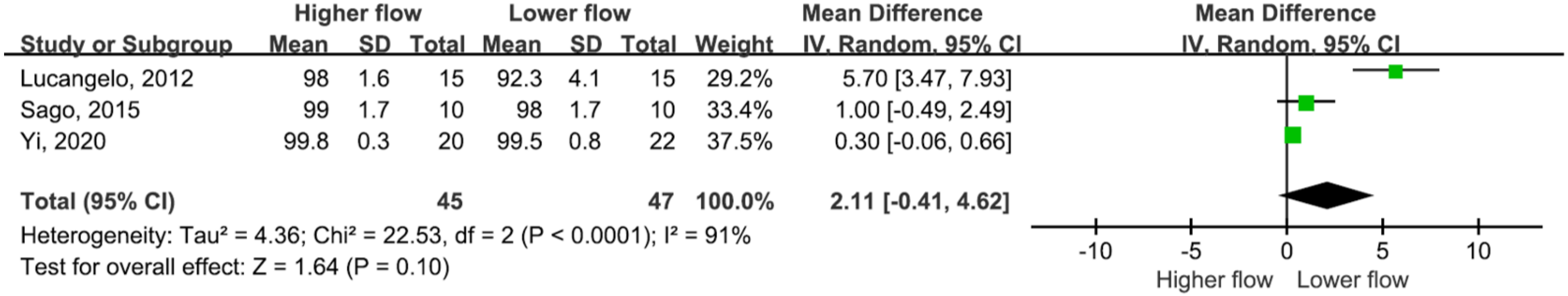

C. PaO_2/F_IO_2

Study or Subgroup	Higher flow Mean	SD	Total	Lower flow Mean	SD	Total	Weight	Mean Difference IV, Random, 95% CI
Lucangelo, 2012	264.4	151.5	15	139.9	22.9	15	47.9%	124.50 [46.96, 202.04]
Yi, 2020	465.4	78	20	475	106.1	22	52.1%	-9.60 [-65.58, 46.38]
**Total (95% CI)**			**35**			**37**	**100.0%**	**54.66 [-76.64, 185.96]**

Heterogeneity: $Tau^2 = 7800.90$; $Chi^2 = 7.55$, $df = 1$ ($P = 0.006$); $I^2 = 87\%$
Test for overall effect: $Z = 0.82$ ($P = 0.41$)

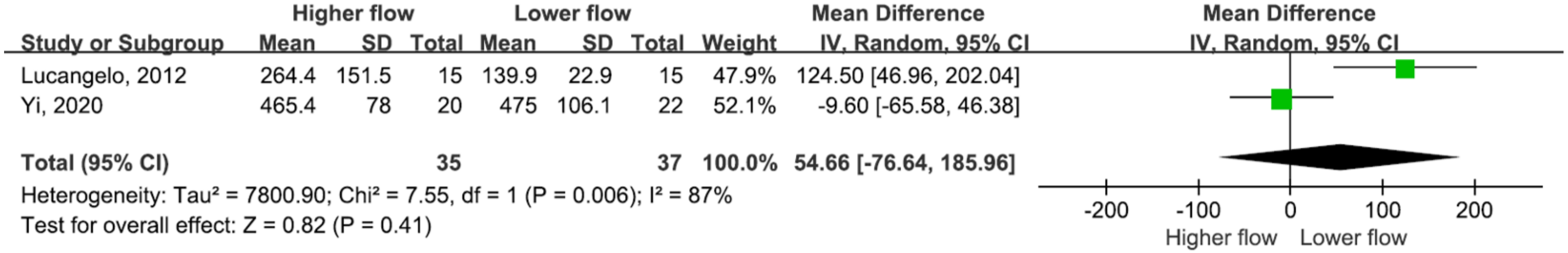

Supplement: Supplementary file 1 — Additional file 1. Assessments of risk of bias for included trials (Figure S1 and Table S1); report of peak tidal inspiratory flow (Table S2); global and regional end-expiratory lung volume with different HFNC flows (Table S3); RCTs (Table S4) and gas exchanges (Figure S2) of different flows during procedural sedation. [file 13054_2023_4361_MOESM1_ESM.pdf]
